# Polyamide monomers *via* carbonate-promoted C–H carboxylation of furfurylamine†

**DOI:** 10.1039/c9sc04460d

**Published:** 2019-11-11

**Authors:** Andrew W. Lankenau, Matthew W. Kanan

**Affiliations:** Department of Chemistry, Stanford University Stanford California 94305 USA mkanan@stanford.edu

## Abstract

Inedible biomass (lignocellulose) is a largely untapped resource for polymer production because it is synthetically challenging to convert to useful monomers. Here we describe streamlined syntheses of two polyamide monomers from furfurylamine, one of very few chemicals made industrially from lignocellulose. Using carbonate-promoted C–H carboxylation, furfurylamine is converted into a furan-containing amino acid and a tetrahydrofuran-containing bicyclic lactam in two and four steps, respectively. Our syntheses avoid the use of protecting groups and multiple stoichiometric organic reagents required by previous, longer routes to these targets. This work facilitates access to furan- and tetrahydrofuran-based polyamides, which are unavailable from petrochemical feedstocks.

## Abstract 2

Carbonate-promoted C–H carboxylation provides a streamlined synthesis of polyamide monomers from a lignocellulosic feedstock without using organic reagents.

## Graphical abstract

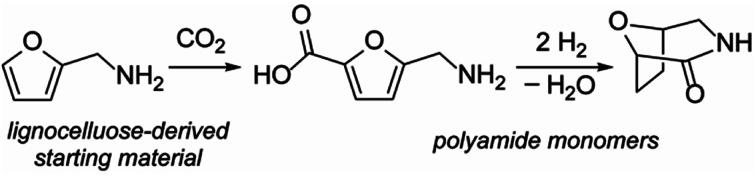

Carbohydrates are attractive feedstocks for polyesters and polyamides because they provide access to monomers that are very difficult to produce from petrochemicals.^1–3^ In addition, sourcing polymers from carbohydrates has the potential to reduce the environmental footprint of plastics provided that land use change is avoided and non-renewable inputs are minimized.^4–6^ The carbohydrate-derived monomers 5-(aminomethyl)-furan-2-carboxylic acid (**1**) and 8-oxa-3-azabicyclo[3.2.1]octan-2-one (**2**) are of interest for the preparation of (tetrahydro)furan-based polyamides (Fig. 1a),^7–10^ but the available syntheses of these monomers are not suitable for polymer applications. In this study, we describe carbonate-promoted C–H carboxylation chemistry that enables streamlined syntheses of **1** and **2** from furfurylamine (**3**), a chemical made industrially by reductive amination of furfural with NH_3_ and H_2_.^11^ Furfural is an attractive starting material because it is prepared by acid-catalyzed depolymerization and dehydration of hemicellulose polymers in raw inedible biomass (lignocellulose).^12^ Sourcing from lignocellulose instead of edible feedstocks makes it possible to avoid land use change by utilizing biomass residues and waste streams.^13,14^

**Fig. 1:**
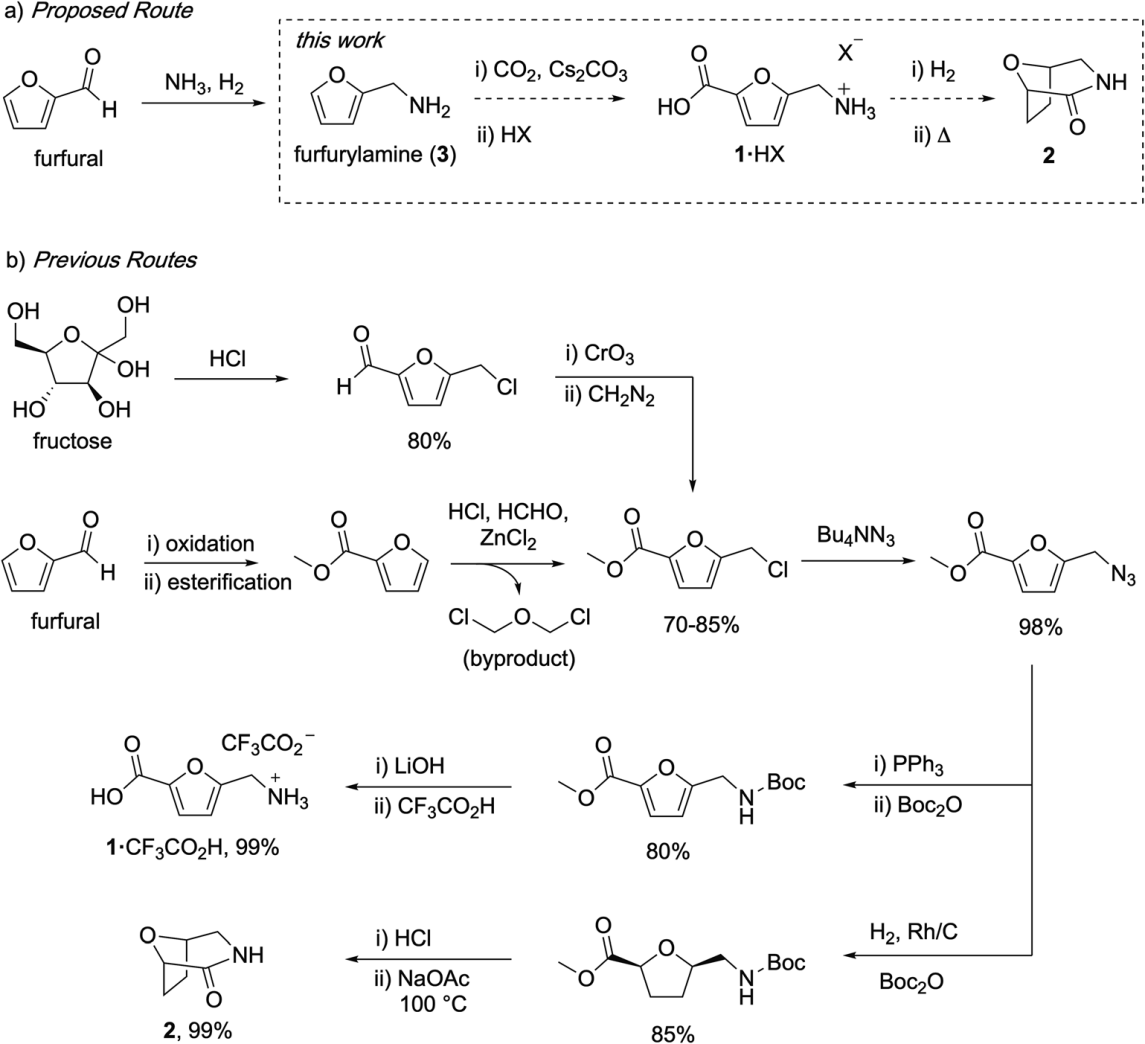
Comparison of the target C–H carboxylation route to **1** and **2** with previous syntheses of these monomers.

Previous syntheses of **1** and **2** start with either fructose or methyl furoate, a commercially available compound made by oxidation and esterification of furfural. Both routes converge on the intermediate methyl 5-(chloromethyl)furan-2-carboxylate and are summarized in Fig. 1b. Synthesizing this intermediate from fructose requires HCl-promoted dehydration to form 5-chloromethylfurfural, Jones oxidation of the aldehyde and subsequent methylation with diazomethane.^15^ Alternatively, methyl 5-(chloromethyl)furan-2-carboxylate is prepared from methyl furoate by Blanc chloromethylation.^7^ To synthesize **1**, the chloromethyl is converted into an azidomethyl functionality by treatment with Bu_4_NN_3_.^16^ Subsequent reduction of the azide with Ph_3_P, Boc protection, ester hydrolysis, and deprotection affords **1** as an ammonium salt.^15^ To synthesize **2**, the azidomethyl intermediate is hydrogenated in the presence of Boc_2_O to afford a Boc-protected aminomethyl tetrahydrofuran intermediate, which is subsequently deprotected and cyclized.^16^ Although the individual steps of these syntheses have good to excellent yields, they consume multiple expensive stoichiometric reagents that do not end up in the final product, generate a toxic waste byproduct (Cr waste or bis(chloromethyl) ether), and utilize a potentially hazardous azide intermediate.

The patent literature reports an alternative synthesis of **1** from 5-chloromethylfurfural using Au-catalyzed alkaline aerobic oxidation and Cl^−^ displacement with liquid NH_3_, but no synthetic details or yields are provided.^8^ Another patent reports a three-step synthesis of **1** from 5-hydroxymethylfurfural that uses superstoichiometric pyridinium tribromide and hydroxylamine, also without yields.^10^ Finally, an alternative enzymatic synthesis of **1** has been reported, but the reaction has a low yield (31%), requires expensive biochemicals and enzyme, and operates at very dilute conditions.^17^

We recently reported that carbonate can promote the carboxylation of very weakly acidic C–H bonds in solvent-free alkali salts at intermediate temperatures under CO_2_.^18,19^ Carbonate-promoted C–H carboxylation can be used to convert furan-2-carboxylate (furoate) into furan-2,5-dicarboxylate in high yield (Fig. 2a), which has opened up a streamlined route from furfural to furan-2,5-dicarboxylic acid (FDCA).^20^ Polyesters based on FDCA have superior properties to conventional terephthalate-based polyesters for numerous high-volume applications,^2,21,22^ which motivates a broader exploration of furan-based monomers. The analogous C–H carboxylation of furfurylamine (**3**) attracted our interest as a way to access to **1** and **2** because it avoids the use of an azide and minimizes the step count. Moreover, the synthesis of **3** from furfural is a highly efficient, single-step process.^11,23^ We hypothesized that C–H carboxylation of **3** could be achieved by converting it into an alkali (furan-2-ylmethyl)carbamate (**4**) and applying conditions used for the carboxylation of alkali furoate (Fig. 2b). In this scheme, the carbamate would serve as a carboxylate surrogate that is readily reverted to an amine by protic decarboxylation.

**Fig. 2:**
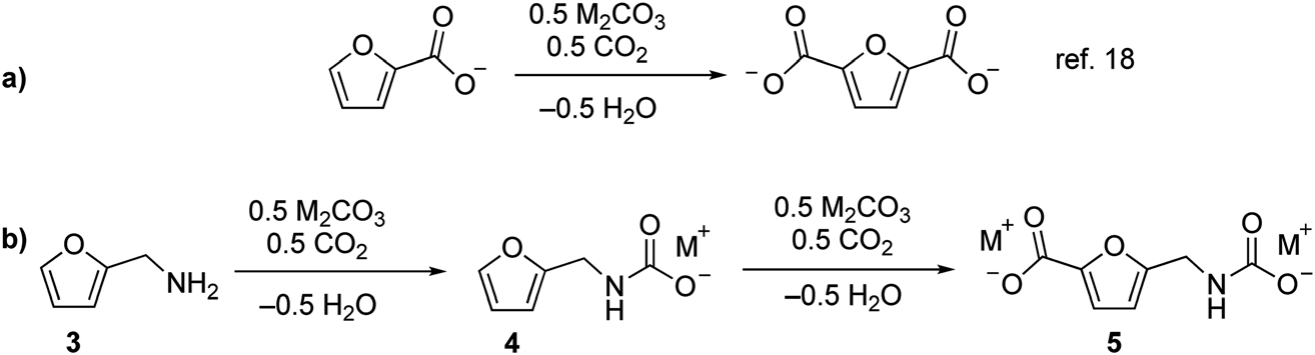
Carbonate-promoted C–H carboxylation of furanics. (a) Carboxylation of furan-2-carboxylate to furan-2,5-dicarboxylate. (b) Conversion of **3** into a carbamate and subsequent C–H carboxylation.

C–H carboxylation was evaluated by combining **3** and M_2_CO_3_ in a Parr reactor and heating this mixture under CO_2_ (Table 1). We first attempted to use flowing CO_2_. Surprisingly, when **3** was combined with an excess of Cs_2_CO_3_ (3.15 equivalents) and heated to 170 °C for 30 min under flowing 1 bar CO_2_, only Cs_2_CO_3_ was recovered. However, when the reaction was performed at 170 °C under static 60 bar CO_2_ for 2 h, 60% yield of carbamate **4**, 26% of desired carboxylated product **5** and 3% recovered **3** was observed (Table 1, entries 1–2). These results indicate that **3** is in equilibrium with **4** when combined with Cs_2_CO_3_ and CO_2_ at elevated temperature. In an open (flowing) system, all of the organic substrate is lost because **3** is removed from the system. However, in a closed system, the desired carboxylation reaction is possible.

**Table 1:** Optimization of furfurylamine C–H carboxylation (1 mmol scale)

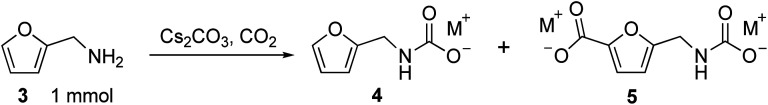							
Entry	*T* (°C)	CO_2_ pressure (bar)	Cs_2_CO_3_ loading (equiv.)	Time (h)	**3** (%)	**4** (%)	**5** (%)
1	170	1 (flowing)	3.15	0.5	0	0	0
2	170	60	3.15	2	3	60	26
3	160	60	3.15	2	4	74	13
4	180	60	3.15	2	2	43	33
5	190	60	3.15	2	2	23	34
6	170	60	3.15	12	1	9	63
7	170	60	1.05	12	3	16	20
8	170	60	6.30	12	0	7	70
9^a^	170^a^	60^a^	3.15^a^	12^a^	1^a^	11 + 3^a^	65 + 5^a^
10	170	30	3.15	12	0	10	61
11	170	10	3.15	12	0	58	20
12	170	10	3.15	24	2	24	19

a A separate vial containing 3.15 equivalents Cs_2_CO_3_ was added to the reactor to serve as a trap for volatile **3**. The second number represents the collected material in the trap.

The reaction was optimized on a 1 mmol scale by evaluating the temperature, pressure, and carbonate loading dependence. Keeping the pressure at 60 bar and the Cs_2_CO_3_ loading at 3.15 equivalents, a screen of reaction temperatures between 160 °C and 190 °C revealed 170 °C to be the optimal temperature (entries 3–5). The reported temperature corresponds to the reading of the internal thermocouple in the Parr (see ESI†).

When the reaction time was increased from 2 h to 12 h at 170 °C, 9% of **4** and 63% of desired product **5** were obtained with only 1% **3** remaining (entry 6). The mass balance of the reaction consisted of **3** that was volatilized and condensed in the reactor head, carbonaceous decomposition products (char), and minor products assigned as di- and tricarboxylates based on NMR characterization (Fig. S1†). In all carboxylation experiments, the combined yield of the over-carboxylated side products was ≤7%.

The carboxylation proved to be sensitive to the Cs_2_CO_3_ loading. Because the CsHCO_3_ that is formed during the reaction can decompose to 0.5 equivalents of H_2_O, CO_2_, and Cs_2_CO_3_, only 1.0 equivalent of Cs_2_CO_3_ is needed, in theory, to convert **3** to **5** (0.5 equivalent for **3** to **4**, 0.5 equivalent for **4** to **5**). However, a much lower yield of **5** was observed when 1.05 equivalents of Cs_2_CO_3_ was used compared to 3.15 equivalents (20% *vs.* 63%, entries 7 and 6, respectively). Increasing the loading to 6.3 equivalents increased the yield of **5** to 70% (entry 8). We hypothesized that the reaction requires superstoichiometric Cs_2_CO_3_ loading to obtain good yields because excess Cs_2_CO_3_ serves as a trap for volatile **3** during the course of the reaction. At lower Cs_2_CO_3_ loadings, large amounts of **3** escape the vial containing Cs_2_CO_3_ and condense elsewhere in the reactor. To test this hypothesis, a reaction was performed by placing two vials in the reactor, the first containing a combination of **3** and 3.15 equivalents Cs_2_CO_3_ and the other containing only Cs_2_CO_3_. At the end of the reaction, 11% of **4** and 65% of **5** were obtained in the vial originally containing **3**, and an additional 3% of **4** and 5% of **5** were observed in the vial initially containing only Cs_2_CO_3_ (entry 9). This experiment confirms that **3** is volatile during the reaction and can be captured by Cs_2_CO_3_.

Our previous studies of furoate carboxylation showed that using blends of Cs_2_CO_3_ and K_2_CO_3_ result in comparable yields as pure Cs_2_CO_3_.^20^ For the carboxylation of **3**, the use of Cs_2_CO_3_ and K_2_CO_3_ blends resulted in greatly diminished yields, indicating that K_2_CO_3_ is not a viable base for this reaction (Table S1†). Similarly low yields were obtained with K_3_PO_4_. Lastly, the effect of pressure was examined. The results at 30 bar CO_2_ were very similar to 60 bar (entry 6 *vs.* entry 10). However, performing the reaction at 10 bar resulted in 58% **4** and only 20% **5** (entry 11). Increasing the reaction time at 10 bar from 12 h to 24 h did not improve the yield of **5** and resulted in more decomposition (entry 12). This result also contrasts with the carboxylation of furoate, in which very high yields can be obtained at <10 bar.^18,20^

To isolate **1** from the crude carboxylation product **5**, we developed a procedure using an ion exchange resin (Fig. 3). Briefly, the crude carboxylation product mixture is dissolved in H_2_O, treated with activated carbon, filtered, and loaded onto a column of strong anion exchange resin that is pre-loaded with HO^−^. The filtrate from the ion exchange column contains CsOH, which makes it straightforward to recycle Cs^+^. In a proof-of-concept, Cs^+^ was recovered in >97% yield from a carboxylation/ion exchange purification sequence (see ESI†). Treatment of the resin loaded with **5** with an aqueous HCl solution effects decarboxylation and elution of aminomethylfuroic acid as an ammonium chloride (**1**·HCl). We note that ion exchange resins are utilized industrially for the purification of several high-volume chemicals, including citric acid.^24,25^

**Fig. 3:**
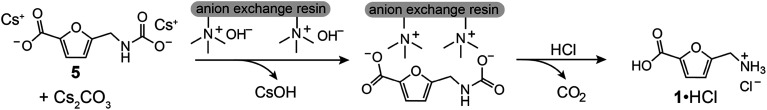
Strategy for isolation of **1** from crude carboxylation product **5**.

The carboxylation reaction was readily increased to the 10 mmol scale with a very similar yield by operating at a nominal reaction temperature of 155 °C for 18 h. Using the ion exchange procedure, **1**·HCl was isolated from this reaction in 62% yield (Fig. 4), which is nearly identical to the crude yield of **5** (Table 1, entry 6). Hydrogenation of **1**·HCl using Rh/C proceeded under mild conditions in H_2_O (25 °C, 10 bar H_2_) to form the *cis* diastereomer with no detectable amount of *trans* (see ESI†), consistent with previous results for the hydrogenation of the Boc-protected aminomethyl derivative of **1**.^16^ The crude hydrogenation product was refluxed in acetic acid and purified by column chromatography to afford pure lactam **2** as white crystals in 76% isolated yield from **1**·HCl.

**Fig. 4:**
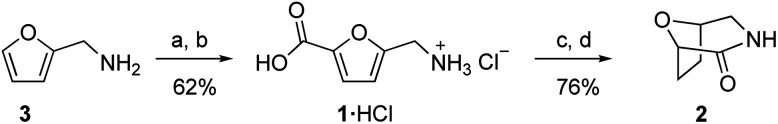
Synthesis of **1**·HCl and **2**. (a) 155 °C, 3.15 equiv. Cs_2_CO_3_, 60 bar CO_2_, 18 h. (b) ion exchange, HCl (10 mmol scale). (c) 0.7 mol% Rh/C, 10 bar H_2_, H_2_O, 25 °C, 2 h. (d) AcOH, reflux, 16 h (3.7 mmol scale).

Our syntheses of **1** and **2** capture several features that are important for scalable monomer production. Given that **3** is produced by hemicellulose dehydration followed by reductive amination, the overall conversion of biomass into **1** and **2** using C–H carboxylation requires only redox-neutral and reductive (hydrogenative) transformations, thereby avoiding unnecessary oxidation state changes. There are no protecting groups or other stoichiometric organic reagents and the only solvents used are H_2_O and acetic acid. The use of Cs_2_CO_3_ necessitates highly efficient Cs^+^ recycling for large-scale application, although it may be possible to reduce the Cs_2_CO_3_ loading and increase the yield by improving the reactor design to avoid volatilization losses.

## Conflicts of interest

The authors declare no competing financial interests.

## Supplementary Material

SC-011-C9SC04460D-s001
