# Supplementary material for: Polyamide monomers via carbonate-promoted C–H carboxylation of furfurylamine
Source: Chem Sci. 2019 Nov 11;11(1):248–52. doi: 10.1039/c9sc04460d (PMC8133028; doi:10.1039/c9sc04460d)
Supplement: SC-011-C9SC04460D-s001 [file SC-011-C9SC04460D-s001.pdf]

Electronic Supplementary Material (ESI) for Chemical Science.
This journal is © The Royal Society of Chemistry 2019

Supplementary Information for

Polyamide Monomers Via Carbonate-Promoted C–H Carboxylation of Furfurylamine

Andrew W. Lankenau[1] and Matthew W. Kanan[1]*

[1]Department of Chemistry, Stanford University, Stanford, California 94305, United States

*Correspondence to: mkanan@stanford.edu

Index	page
**Experimental Methods**	S2-S4
**Table S1**. Carboxylation of furfurylamine (**3**) using various alkali bases	S5
**Figure S1**. Representative crude ^{1}H NMR spectrum of furfurylamine (**3**) carboxylation	S6
**Figure S2**. Representative ^{1}H and ^{13}C NMR spectra of isolated 5-(aminomethyl)furan-2-carboxylic acid hydrogen chloride (**1**·HCl)	S7
**Figure S3**. Representative ^{1}H and ^{13}C NMR spectra of isolated (±)-*cis*-5-(aminomethyl)tetrahydrofuroic acid hydrogen chloride	S8
**Figure S4**. Representative ^{1}H and ^{13}C NMR spectra of isolated (±)-8-oxa-3-azabicyclo[3.2.1]octan-2-one (**2**)	S9

Experimental Methods

Chemicals and materials.

Cesium carbonate (>99.9%, trace metal basis) was purchased from Chem Impex International; D_2O (99.9%) was purchased from Cambridge Isotope Laboratories; furfurylamine (>99%) was purchased from Sigma-Aldrich; anhydrous potassium carbonate (99.9%), glacial acetic acid, concentrated hydrochloric acid, and sodium L-(+)-tartrate dehydrate (99.7%) were purchased from Fisher Scientific; carbon dioxide (99.5%) was purchased from Praxair; rhodium (5%) on activated wood carbon, reduced, 50% water wet paste was purchased from Strem Chemicals; Activated Carbon Norit SA 2 and Celite 545 were purchased from Acros Organics. For small scale reactions (1.0 mmol), all chemicals were used as received without further purification. For large scale (10 mmol) carboxylation reactions, furfurylamine was freshly distilled with calcium hydride and stored under nitrogen.

General Methods.

NMR spectra were obtained at 23 °C on either a 600, 500, or 400 MHz Varian Unity Inova spectrometer. All chemical shifts are reported in ppm and are referenced to tetramethylsilane using the ^1H or ^{13}C signals of methanol (^1H chemical shift $\delta = 3.34$, ^{13}C chemical shift $\delta = 49.00$), chloroform (^1H chemical shift $\delta = 7.26$, ^{13}C chemical shift $\delta = 77.16$) or D_2O (^1H chemical shift $\delta = 4.79$) as internal standards. Coupling constants (J) are reported in hertz (Hz). Deuterated solvents were used as received from Cambridge Isotopes. Pressurized CO_2 experiments were performed in a 300 mL high-temperature, high-pressure stainless steel Parr reactor (model 4561-HT-FG-SS-115-VS-2000-4848) equipped with a glass liner. Pressurized H_2 experiments were performed in a 250 mL high-temperature, high-pressure hastelloy Parr reactor (model 4576B-FG-M(HB)-HB-115-VS.12-50000-AS-4848-PDM-MCM-HTM-A1925E4-PARR-4579D). High-resolution mass spectra were recorded on a Waters Acquity UPLC and Thermo Exactive Orbitrap mass spectrometer by direct injection electrospray ionization-mass spectrometry (ESI-MS).

General procedure for furfurylamine (**3**) carboxylation to alkali 5-((carboxylatoamino)methyl)furan-2-carboxylate **5** (1 mmol).

To a 20 mL glass vial was added furfurylamine (0.097 g, 1.0 mmol) followed by anhydrous alkali carbonate (various loadings). The vial was capped with a septa and CO_2 was flowed through the vial (40 sccm) at room temperature for 15 minutes. The septa was removed and the vial was placed into the Parr reactor and sealed. The reactor was placed under vacuum, then filled to ~1 bar CO_2. This cycling process was repeated five times. The reactor was then filled to a final CO_2 pressure at ambient temperature corresponding to a pressure of either 10, 30, or 60 bar at the desired reaction temperature. The reactor was then heated to the desired temperature, maintained for the desired time, cooled, and depressurized. The reported reaction temperature corresponds to the temperature recorded by a J-type thermocouple height-adjusted by Parr such that the tip lies just above the reactor floor. The thermocouple does not directly measure the temperature of the solid reactant mixture, which is inevitably subjected to thermal gradients. The crude product in the vial was dissolved in D_2O containing an internal tartrate standard and quantified using ^1H NMR spectroscopy. See **Figure S1** for a representative crude ^1H NMR spectrum.

Synthesis and isolation of 5-(aminomethyl)furan-2-carboxylic acid hydrogen chloride (**1**·HCl).

To a 300 mL glass liner was added freshly distilled furfurylamine (0.971 g, 10.0 mmol) followed by anhydrous cesium carbonate (10.263 g, 31.5 mmol). The liner was placed into the Parr reactor and sealed. The reactor was pressurized with CO_2 to ~12 bar then vented to ~2 bar. This process was repeated five times before being pressurized to ~39 bar CO_2. The reactor was heated to 155 °C and reached a reaction pressure of ~60 bar. After heating at 155 °C for 18 hours, the reactor was cooled. The brown/black crude material was dissolved in 25 mL deionized H_2O, treated with ~2 g activated carbon, and filtered through a 3 cm celite plug in a 60 mL medium frit yielding a homogeneous, pale orange solution. Next, a 20 cm column (approx. 80 g) of anion-exchange resin Amberlite IRA-400(Cl^-) was washed with 400 mL 1 M HCl, then deionized H_2O until the filtrate was pH neutral, then 400 mL 1 M NaOH, and then deionized H_2O until the filtrate was pH neutral. This washing procedure was repeated once more to remove manufacturing residues on the resin. After washing, the filtered reaction solution was loaded onto the column and slowly eluted. Deionized H_2O was added to the column until the eluent was pH neutral. Afterwards, the product was removed from the column by washing three times with 200 mL 1 M HCl. Bubbling was observed due to decarboxylation of the product carbamate and adsorbed carbonate/bicarbonate. The clear, homogeneous filtrate was evaporated to a residue under reduced pressure. The residue was redissolved in a mixture of 200 mL isopropanol and 10 mL deionized H_2O and evaporated to approximately 40 mL, during which a thick white precipitate was observed. To this heterogeneous solution was added 200 mL diethyl ether and the white precipitate was filtered over 30 mL medium frit and washed with 25 mL diethyl ether. The collected material was transferred to a vial and placed under vacuum overnight to dry (1.099 g, 6.188 mmol, 62 % yield). ^{1}H NMR (D_2O, δ): 7.28 (1H, d, *J* = 3.6), 6.72 (1H, d, *J* = 3.6), 4.31 (2H, s). ^{13}C NMR (D_2O, δ): 161.8, 150.7, 145.3, 119.9, 113.2, 35.8. ESI-MS $C_6H_8NO_3^+$ observed, calculated: 142.0497, 142.0499; 143.0530, 143.0529. ^{1}H and ^{13}C NMR spectra are shown in **Fig. S2**

Synthesis and isolation of crude (±)-*cis*-5-(aminomethyl)tetrahydrofuran-2-carboxylic acid hydrogen chloride.

To a 20 mL glass vial was added 5-(aminomethyl)furan-2-carboxylic acid hydrogen chloride (0.444 g, 2.5 mmol), 5 wt% wet rhodium on carbon (0.130 g, 0.032 mmol Rh), and deionized H_2O (10 mL). The solution was mixed, placed into a Parr reactor equipped with a stirring rod, and sealed. The reactor was pressurized with N_2 to ~10 bar and vented to ~2 bar five times. The reactor was then pressurized with H_2 to ~12 bar and vented to ~2 bar five times before being pressurized to 10 bar H_2. The stirrer was turned on to a rotation rate of 450 rpm, and the reactor was kept at room temperature for 4 h. The heterogeneous solution was filtered over a 2 cm celite plug in a 30 mL medium frit and washed with ~50 mL deionized H_2O. The filtrate was evaporated to a residue and used without further purification (0.490 g, 81% pure, 2.186 mmol, 88% yield). ^{1}H NMR (D_2O, δ): 4.58 (1H, dd, *J* = 8.7, 4.9), 4.40 (1H, m), 3.27 (1H, dd, *J* = 13.4, 2.6), 3.13 (1H, dd, *J* = 13.4, 7.6), 2.42 (1H, m), 2.15 (2H, m), 1.74 (1H, m). ^{13}C NMR (D_2O, δ): 178.5, 77.5, 76.8, 42.2, 29.9, 27.3. ESI-MS $C_6H_{12}NO_3^+$ observed, calculated: 146.0810, 146.0812; 147.0842, 147.0843. ^{1}H and ^{13}C NMR spectra are shown in **Fig. S3**.

Hydrogenation and sequential cyclization of 5-(aminomethyl)furan-2-carboxylic acid hydrogen chloride (**1**·HCl) to (±)-8-oxa-3-azabicyclo[3.2.1]octan-2-one (**2**).

To a Parr reactor equipped with a stirring rod was added 5-(aminomethyl)furan-2-carboxylic acid hydrogen chloride (0.664 g, 3.74 mmol), 5 wt% wet rhodium on carbon (0.102 g, 0.025 mmol Rh), and deionized H_2O (30 mL). The reactor was sealed pressurized with N_2 to ~10 bar and vented to ~2 bar five times. The reactor was then pressurized with H_2 to ~12 bar and vented to ~2 bar five times before being pressurized to 10 bar H_2. The stirrer was turned on to a rotation rate of 250 rpm, and the reactor was kept at room temperature for 2 hours. Afterwards, the heterogeneous solution was passed through a 0.2 µm PVDF syringe filter, evaporated to a residue in a 500 mL roundbottom flask, and placed under vacuum overnight. The next day, glacial acetic acid (120 mL) and a magnetic stir bar were added to the roundbottom flask. The flask was then connected to a condenser open to air and refluxed for 16 h. After cooling, the solvent was evaporated to a thick yellow oil. The oil was dissolved in 10 mL 95:5 dichloromethane:methanol, filtered over a 2 cm celite plug in a 15 mL frit, and washed with an additional 50 mL 95:5 dichloromethane:methanol. The filtrate was then evaporated to a residue. The crude product was purified by silica gel column chromatography by eluting with 95:5 dichloromethane:methanol, affording a white crystalline solid (0.361 g, 2.84 mmol, 76% yield). ^{1}H NMR ($CDCl_3$, δ): 5.99 (1H, br s), 4.62 (1H, m), 4.46 (1H, d, J = 7.1), 3.69 (1H, dd, J = 11.2, 4.1), 3.01 (1H, d, J = 11.3), 2.24-2.10 (3H, m), 1.88 (1H, m). ^{13}C NMR ($CDCl_3$, δ): 172.5, 77.3, 72.1, 48.3, 31.9, 28.0. ESI-MS $C_6H_{10}NO_2^+$ observed, calculated (%): 128.0704, 128.0706; 129.0737, 129.0737.

Recovery of cesium via formation of cesium chloride.

The general procedure for the synthesis and isolation of **1**·HCl (see 'Synthesis and isolation of 5-(aminomethyl)furan-2-carboxylic acid hydrogen chloride') was followed using the following quantities of furfurylamine (0.971 g, 10.0 mmol) and anhydrous cesium carbonate (10.236 g, 31.4 mmol). Once the reaction had completed and cooled to room temperature, the resulting solid was dissolved in 25 mL deionized H_2O, treated with ~2 g activated carbon, and filtered through a 3 cm celite plug in a 60 mL coarse frit. Next, a 36 cm column of anion-exchange resin Amberlite IRA-400(Cl^-) was washed with 400 mL 1M HCl, then deionized H_2O until the filtrate was pH neutral, then 400 mL 1M NaOH, and then deionized H_2O until the filtrate was pH neutral. This washing procedure was repeated once more to remove manufacturing residues on the resin. After washing, the filtered reaction solution was loaded onto the column, slowly eluted, and collected. Deionized H_2O was added to the column, eluted until the pH was neutral, and also collected. The combined eluents, consisting of dilute CsOH, was evaporated to a thick white slurry before being placed in an ice bath. 100 mL 1 M HCl was then slowly added, yielding a clear homogeneous solution. The solution was removed from the ice bath, evaporated to a residue, and placed under vacuum overnight to dry yielding a white solid (10.264 g, 61.0 mmol, >97% cesium recovery)

Table S1. Carboxylation of furfurylamine (3) using various alkali bases

Entry	Salt composition (eq)	T (°C)	**3** (%)	**4** (%)	**5** (%)
1	3.15 Cs_2CO_3	170	1	9	63
2	1.05 Cs_2CO_3, 4.95 K_2CO_3	170	1	34	35
3	3.15 K_2CO_3	190	1	22	0
4	3.15 K_2CO_3, 1.0 K^+isobutyrate	190	2	26	3
5	3.15 K_2CO_3, 1.0 K^+acetate	190	1	22	5
6	1.05 Cs_2CO_3, 3.22 K_3PO_4	170	0	36	23
7	4.82 K_3PO_4	170	12	57	8

Conditions: 1.0 mmol **3**, 60 bar CO_2, 12 hours

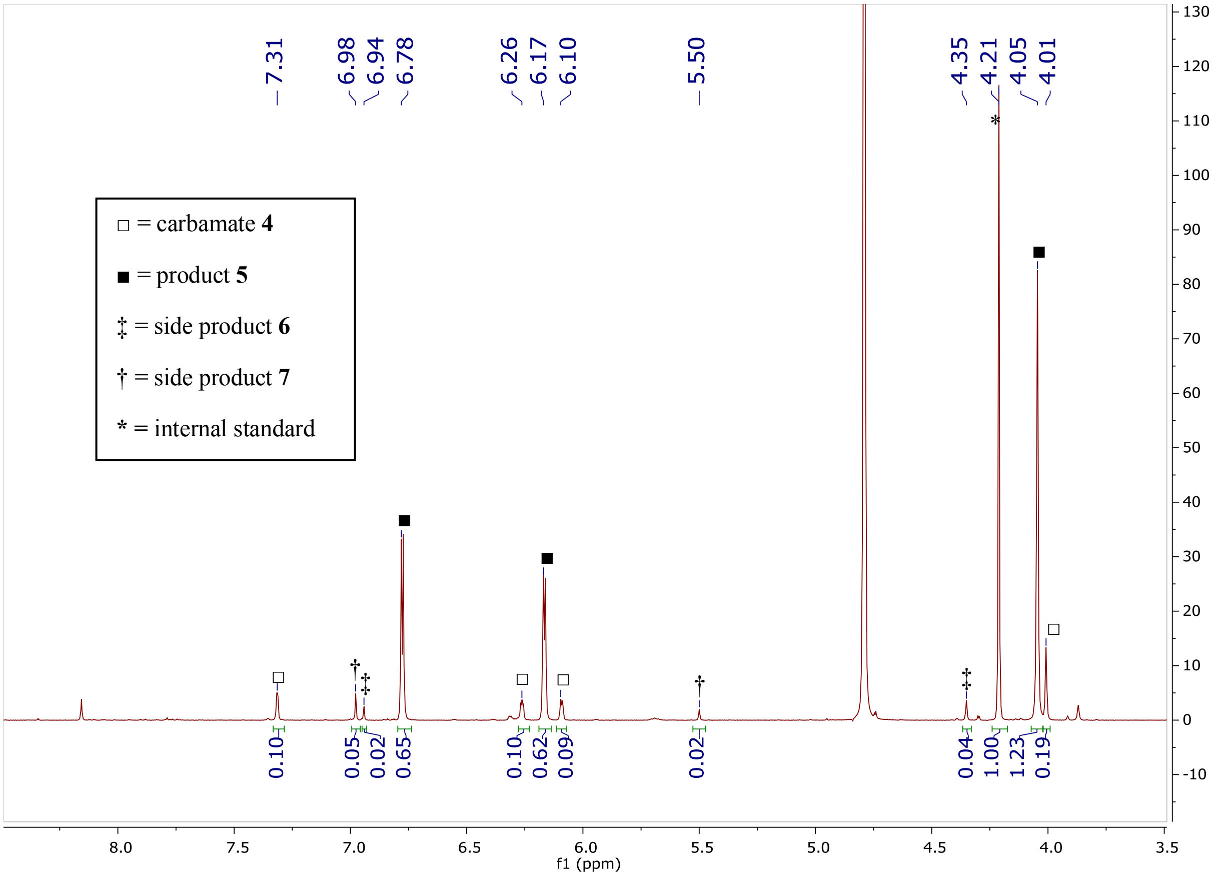

Figure S1: ^{1}H NMR (400 MHz) of the crude product mixture in D_2O after reaction of 1 mmol furfurylamine (**3**) with 3.15 mmol Cs_2CO_3 under 60 bar CO_2 at 170 °C for 12 h. Resonances are referenced to D_2O.

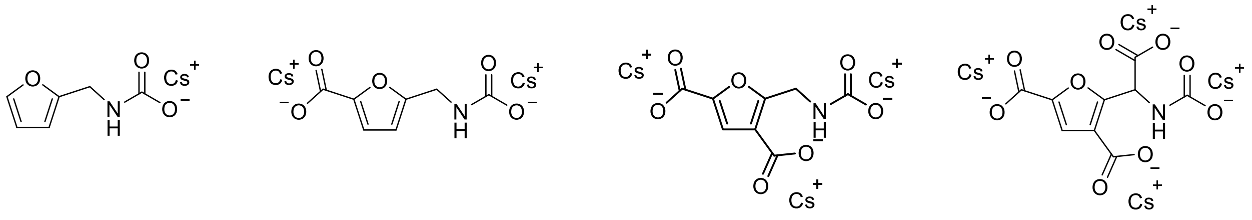

4 **5** **6** **7**

The ^{1}H resonances for cesium (furan-2-ylmethyl)carbamate (**4**), the carboxylated product cesium 5-((carboxylatoamino)methyl)furan-2-carboxylate (**5**), and the side products cesium 5-((carboxylatoamino)methyl)furan-2,4-dicarboxylate (**6**) and cesium 5-(carboxylato(carboxylatoamino)methyl)furan-2,4-dicarboxylate (**7**) are labeled. Note the methine proton of **7** at 5.50 ppm exchanges with D_2O in the basic aqueous solution, accounting for its low integration.

a)

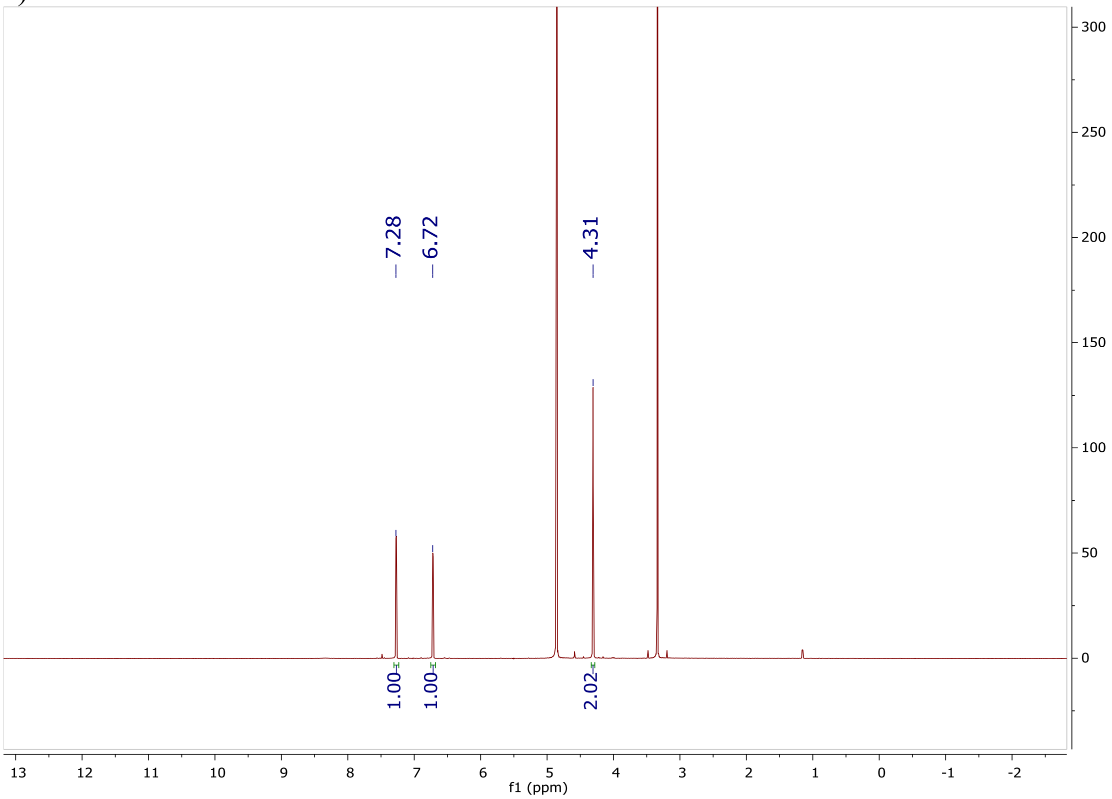

b)

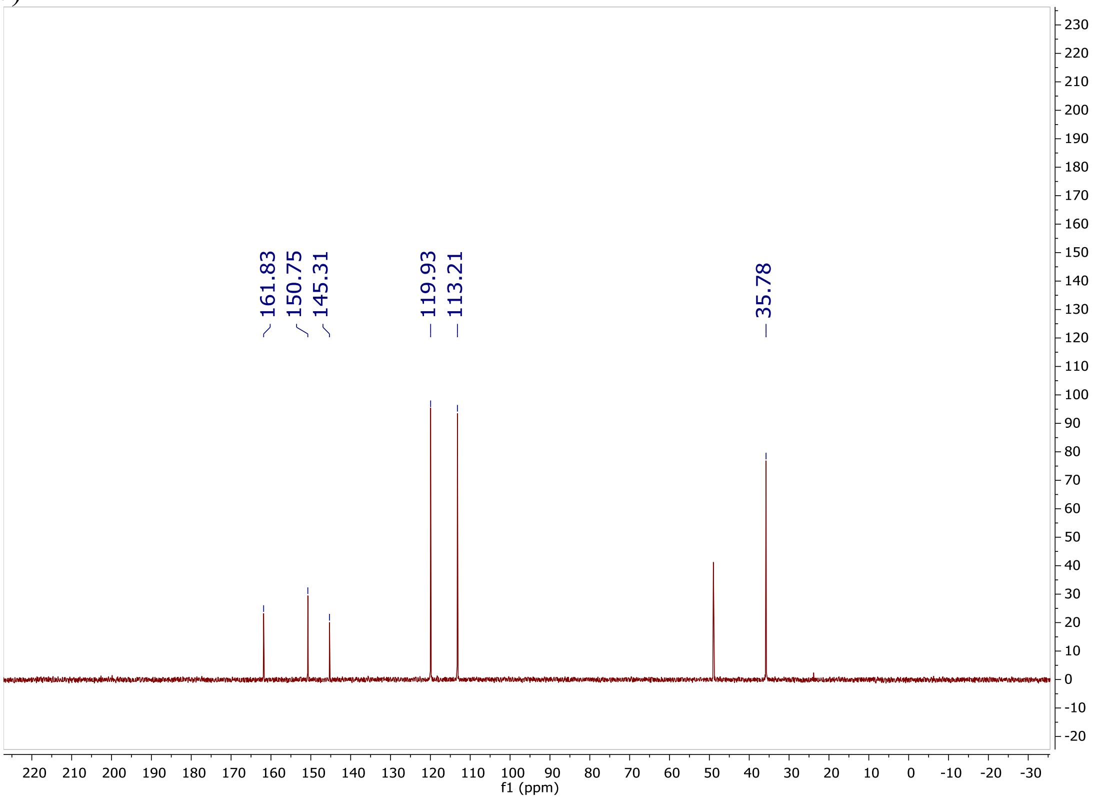

Figure S2: a) ^{1}H NMR (500 MHz) and b) ^{13}C NMR (125 MHz) in D_2O of isolated 5-(aminomethyl)furoic acid hydrogen chloride (**1**·HCl). Resonances are referenced to MeOH.

a)

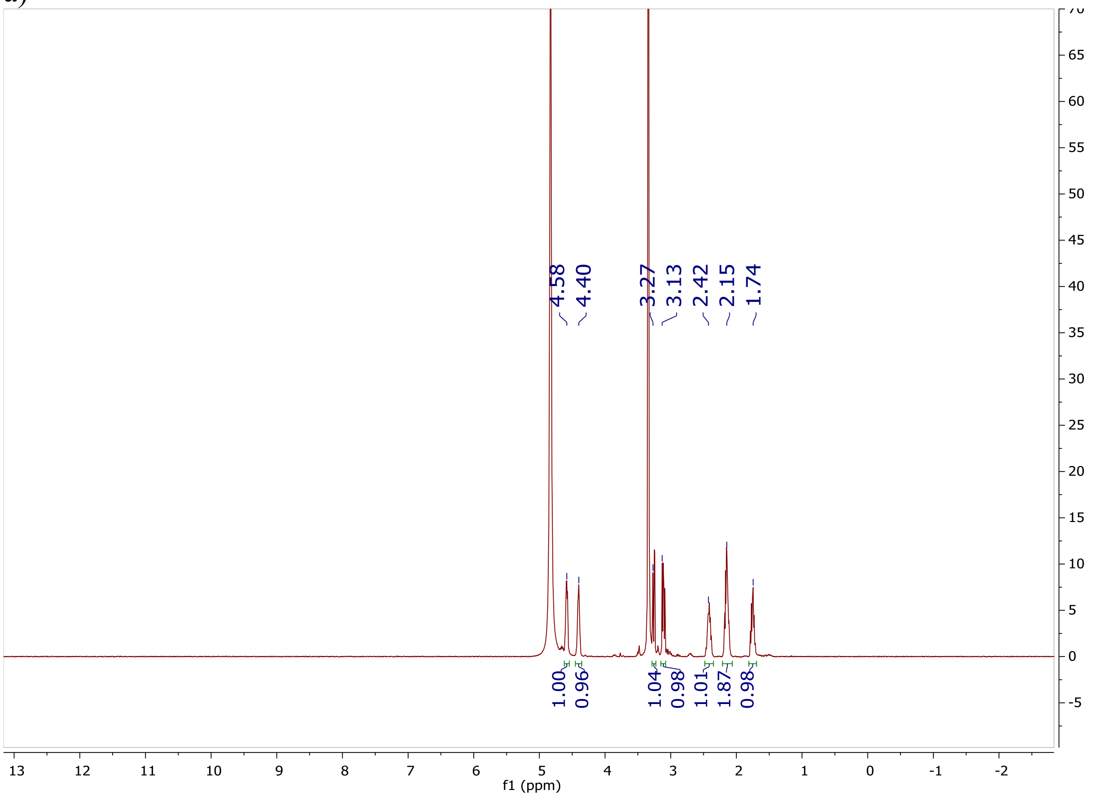

b)

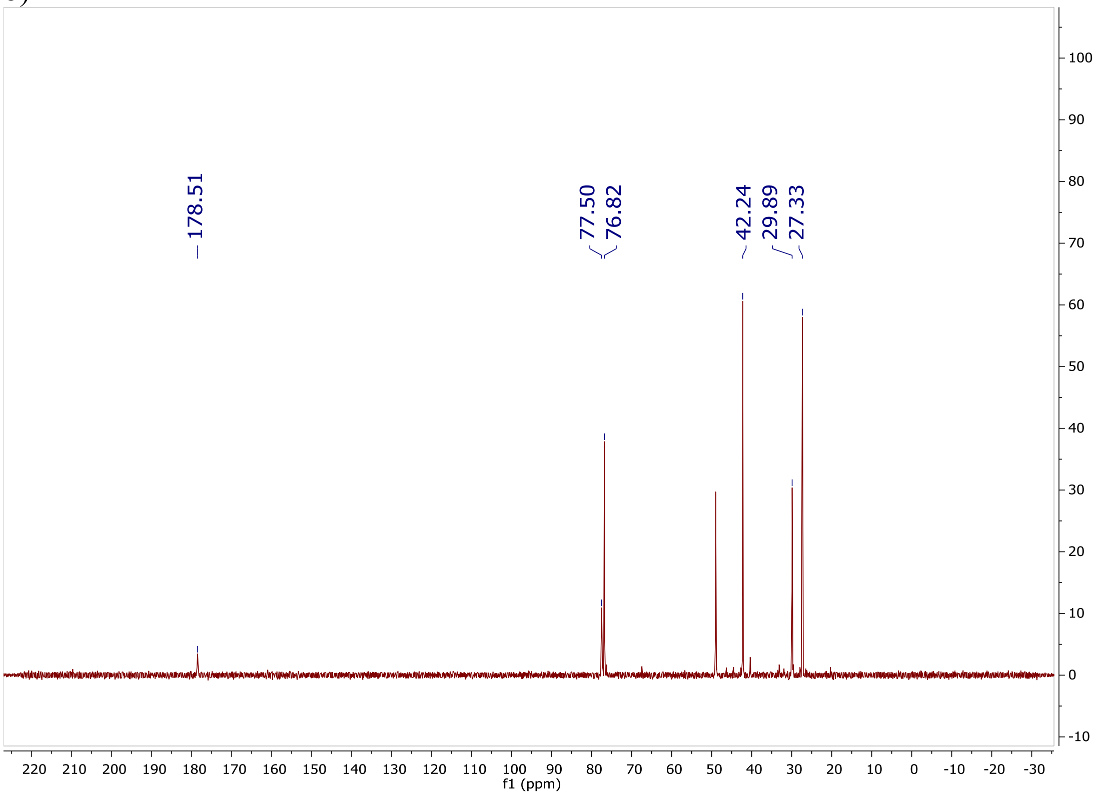

Figure S3: a) ^{1}H NMR (500 MHz) and b) ^{13}C NMR (125 MHz) in D_2O of (±)-*cis*-5-(aminomethyl)tetrahydrofuroic acid hydrogen chloride. Resonances are referenced to MeOH.

a)

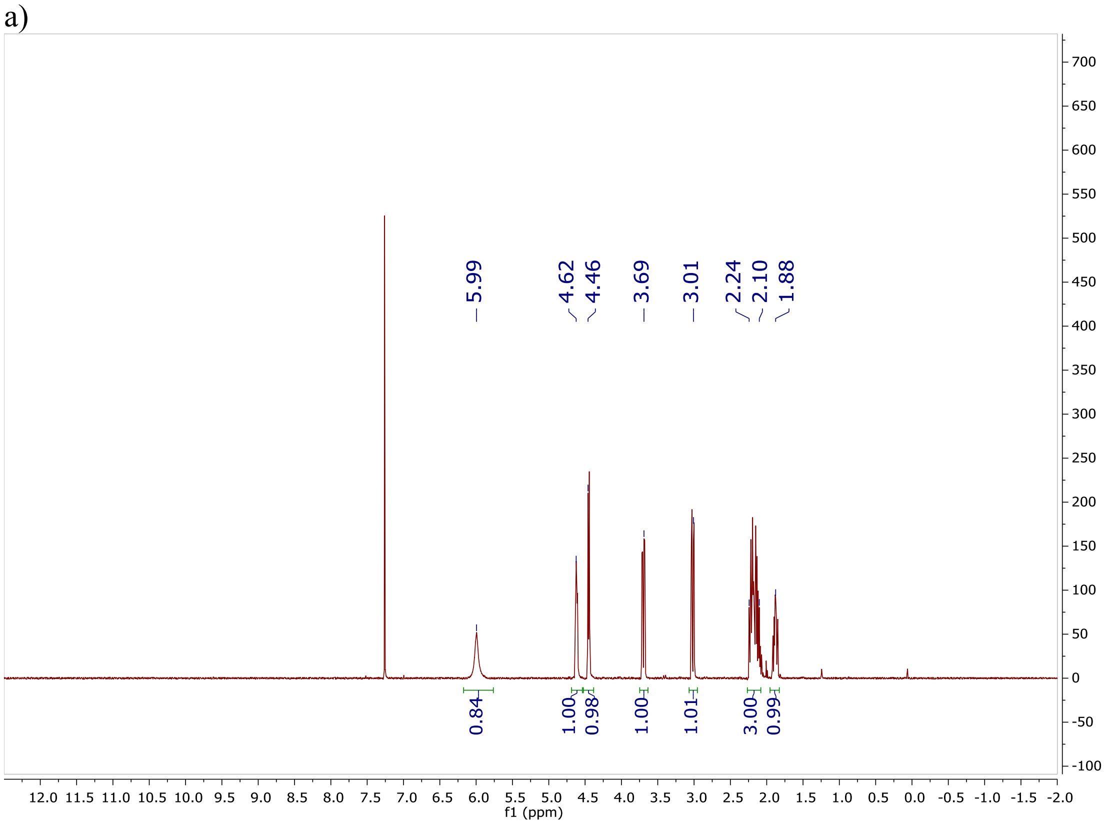

b)

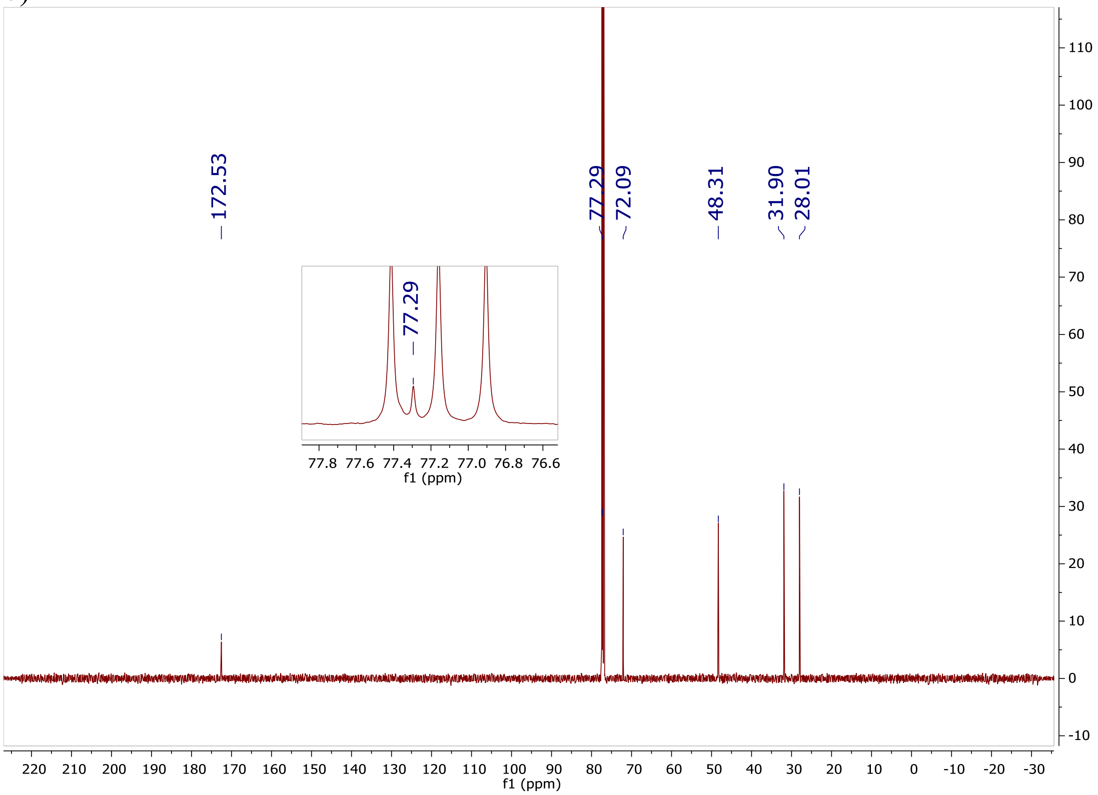

Figure S4: a) ^{1}H NMR (500 MHz) and b) ^{13}C NMR (125 MHz) in $CDCl_3$ of (±)-8-oxa-3-azabicyclo[3.2.1]octan-2-one (**2**). Resonances are referenced to $CHCl_3$.
